# OMICS Analyses in Xenotransplantation: Initial Findings, Key Precautions, and Virus Infections

**DOI:** 10.1111/xen.70095

**Published:** 2025-11-26

**Authors:** Joachim Denner

**Affiliations:** ^1^ Institute of Virology Free University Berlin Berlin Germany

**Keywords:** OMICS, rejection, viral infections, xenotransplantation

## Abstract

OMICS analyses have the potential to greatly enhance our understanding of rejection and other processes in xenotransplantation. These approaches may therefore contribute to extending the survival time of xenotransplants. Initial OMICS studies in brain‐dead patients following transplantation of pig kidneys or hearts revealed increased expression of genes associated with humoral immune responses. This included activation of monocytes, macrophages, and natural killer (NK) cells, as well as endothelial activation, complement activation, and T cell development. Such multimodal deep phenotyping has been proposed as a potential game‐changer in the field of xenotransplantation. However, it is essential to consider that viral infections may significantly influence the results of these analyses. Viruses are known to alter gene expression patterns, not only within the immune system but also in endothelial cells and other tissue compartments. Depending on the type of virus, the immune response may either be stimulated—as the host reacts against the infection—or suppressed, in cases involving immunosuppressive viruses. Therefore, integration of comprehensive virus screening is essential in such studies.

## 1 Introduction

OMICS analyses refer to the comprehensive investigation of all relevant molecules within biological systems. Depending on the type of molecules studied, the field is divided into various sub‐disciplines, including genomics, transcriptomics, proteomics, metabolomics, metagenomics, and phenomics. Compared to traditional molecular techniques such as polymerase chain reaction (PCR) or real‐time PCR, which detect individual genes, reverse transcriptase PCR, which measures gene expression at the ribonucleic acid (RNA) level, or Western blot analysis and immunohistochemistry, measuring expression at the protein‐level, methods like OMICS approaches provide a much broader and more integrative view of biological processes. Additionally, interactomics focuses on mapping the interactions between molecules, offering further insights into the complex networks that govern cellular function.

In the field of xenotransplantation, the first comprehensive OMICS investigations have recently been conducted. These included studies of pig kidneys transplanted into brain‐dead human recipients, multi‐omics profiling in human decedents who received pig heart transplants, and the first pig heart transplantation to a human patient.

## 2 First OMICS Investigations in Xenotranslantation

Loupy et al. [1] conducted comprehensive phenotyping of two pig kidney xenotransplants in human decedents using a multimodal approach. This strategy combined morphological assessment, immunophenotyping, gene expression profiling, whole‐transcriptome digital spatial profiling, and cell deconvolution. As controls, the study included genetically modified pig kidneys prior to implantation, wild‐type pig kidney autotransplants, and wild‐type, non‐transplanted pig kidneys both with and without ischemia‐reperfusion injury.

The transplanted pig kidneys exhibited increased expression of genes associated with humoral immune responses, including activation of monocytes, macrophages, and natural killer (NK) cells, as well as endothelial activation, complement activation, and T‐cell development. Whole‐transcriptome digital spatial profiling revealed a significant enrichment of transcripts linked to monocytes, macrophages, neutrophils, and NK cells, predominantly localized in the glomeruli of the transplanted kidneys. These changes were absent in the control kidneys.

In a study of porcine cardiac xenotransplantation in brain‐dead human recipients, hearts from cloned pigs with 10 distinct genetic modifications were used. These source animals featured a triple knockout of GGTA1, CMAH, and B4GALNT2/B4GALNT2L, deletion of the growth hormone receptor, and transgenic expression of human CD46, CD55, CD59, thrombomodulin, endothelial protein C receptor, and heme oxygenase [2]. To investigate cellular and molecular responses, bulk and single‐cell transcriptomics, lipidomics, proteomics, and metabolomics on blood samples were performed, along with histological and transcriptomic analyses of the xenogeneic heart tissue [3]. Given that longitudinal multi‐omics profiling has proven to be a powerful tool for identifying novel biomarkers, blood samples were collected from the transplanted decedents every 6 h. At 42 h post‐transplantation, substantial alterations in cellular metabolism and liver injury–related pathways were observed in Decedent 1. In contrast, Decedent 2 exhibited relatively modest changes across RNA, protein, lipid, and metabolic profiles. Despite this interindividual variation, the multi‐omics analyses successfully delineated distinct responses to cardiac xenotransplantation and offered novel insights into early molecular and immune events following xenotransplantation in humans.

OMICS analyses have also been conducted in the case of the first pig heart transplant recipient. Single‐cell RNA sequencing revealed changes in immune cell composition, along with increased expression of genes previously associated with allotransplant rejection, inflammation, and interferon responses [4]. However, it remains unclear whether these gene expression changes can be attributed to xenotransplant rejection or to infection with porcine cytomegalovirus transmission—which is actually a porcine roseolovirus (PCMV/PRV). A major achievement of the study was the successful detection of PCMV/PRV infection through analysis of plasma microbial cell‐free DNA (cfDNA) using the Karius test [5]. This assay employs next‐generation sequencing to identify more than 1000 pathogens [6].

Recently, Reichart et al. [7] reviewed advancements in multimodal deep phenotyping, emphasizing that, alongside histopathology and immunophenotyping, gene expression analyses can provide valuable insights into immune responses against xenotransplants. Notably, their contribution was published in the “Game Changer” section of the journal *Transplantation*, highlighting the transformative potential of this integrative approach.

## 3 Necessary Precautions

Unfortunately, some of these studies and commentaries have overlooked a critical factor: the infection status of both the donor organ and the recipient. Infections can profoundly influence gene expression—not only in activated immune cells but also in epithelial and other tissue compartments.

In the kidney transplantations into human brain‐dead individuals, the absence of porcine endogenous retrovirus (PERV) transmission, as well as the absence of seven other porcine viruses in the recipients, has been demonstrated [2, 8]. However, the porcine virome comprises hundreds of viruses (for review, see [9]) that were not tested, and their potential for transmission remains unknown. These viruses may induce immune responses or interact with recipient cells, altering gene expression even in the absence of clinical symptoms. It is important to note that when analyzing the porcine virome using next‐generation sequencing (NGS), viruses with known or suspected zoonotic potential—although frequently detected by PCR‐based or immunological methods—have rarely been identified by NGS. Their detection therefore appears to require more sensitive methods [9]. Moreover, baboon or human viruses present in the transplant recipient may become reactivated, as shown after transplantation of pig thymokidneys into baboons [10], potentially influencing gene expression and immune responses.

The impact of PCMV/PRV has been well studied in xenotransplanted non‐human primates [11]. In baboons that received PCMV/PRV‐positive pig heart xenotransplants, a marked increase in interleukin‐6 (IL‐6) and tumor necrosis factor (TNF) levels were observed [11]. In contrast, serum levels of interferon‐γ (IFN‐γ), IL‐2, IL‐4, IL‐5, and IL‐10 remained unchanged. Additionally, alterations in the coagulation system were detected, including elevated levels of tissue plasminogen activator (tPA) and tPA–plasminogen activator inhibitor‐1 complexes (tPA–PAI‐1) [11].

PCMV/PRV is an immunosuppressive virus, modulating immune gene expression in infected pigs. Specifically, it downregulates pro‐inflammatory cytokines such as IL‐8 and TNF‐α, while upregulating the anti‐inflammatory cytokine IL‐10 in macrophages [12]. In a microarray‐based transcriptomic analysis of thymic tissue from PCMV/PRV‐infected pigs, a total of 5582 genes were differentially expressed compared to uninfected controls—2161 genes were upregulated, and 3421 were downregulated. Selected findings were validated using quantitative real‐time RT‐PCR and Western blot analysis.

Further analysis through gene ontology, gene interaction networks, and KEGG pathway mapping revealed that PCMV/PRV affects multiple functional pathways, including those involved in the immune system, cellular and metabolic processes, cytokine–cytokine receptor interactions, the TGF‐β signaling pathway, the lymphocyte receptor signaling pathway, and the TNF‐α signaling pathway [12].

Additional studies using porcine macrophages and microRNA profiling confirmed that PCMV/PRV infection leads to significant modulation of immune‐regulatory genes [13]. In vitro infection of monocyte‐derived macrophages (MDMs) resulted in decreased mRNA expression of IL‐8 and TNF‐α, and increased expression of IL‐10, further supporting the virus's immunosuppressive profile [14].

Several OMICS‐based studies have also been conducted in humans infected with various viruses, including HIV [15, 16, 17], SARS‐CoV‐2 [18], influenza virus [19], adenovirus [20], and others.

HIV is a well‐characterized immunosuppressive virus that significantly alters cytokine expression in infected individuals. Studies have shown that levels of TNF‐α, IFN‐γ, IL‐1β, IL‐6, and IL‐10 are elevated in HIV‐1–positive individuals, while IL‐17 tends to be lower compared to healthy controls [15]. Notably, even HIV‐positive adults with undetectable viral loads and restored CD4+ T cell counts due to long‐term antiretroviral therapy (ART) continue to exhibit elevated levels of these cytokines [15]. Among these, IL‐10 and TNF‐α are particularly associated with disease progression. While highly active antiretroviral therapy (HAART) induces a gradual reduction in IL‐10 levels, it does not fully normalize them [16].

In addition, transcriptomic profiling of CD4^+^ T cells from HIV‐1–infected patients has revealed that elevated oxidative phosphorylation (OXPHOS) is linked to poor clinical outcomes. The mitochondrial innate immune receptor NLRX1 is also significantly upregulated in these cells [13]. Complementary quantitative proteomic and metabolic analyses demonstrate that NLRX1 enhances both OXPHOS and glycolysis during HIV‐1 infection, thereby facilitating viral replication.

Analysis of plasma samples from 117 individuals with long COVID revealed persistent alterations in cytokine levels, the proteome, and the metabolome. The findings indicated sustained inflammatory responses, platelet degranulation, and cellular activation, alongside significant metabolic dysregulation involving arginine biosynthesis, methionine and taurine metabolism, and tricarboxylic acid (TCA) cycle pathways [18].

Single‐cell RNA sequencing of peripheral blood mononuclear cells (PBMCs) from individuals infected with seasonal influenza A H1N1 virus revealed upregulation of the chemokine CCL2 compared to healthy controls [19]. Similarly, lung tissues from H1N1‐infected mice showed increased expression of interferon‐stimulated genes, including Ifit3, Ifit1, and Isg15. Transcription factor analysis identified a conserved upregulation of key antiviral regulons, such as STAT1 and IRF7, in T cells across both species. These findings indicate that humans and mice share common immune responses to H1N1 infection, highlighting the conservation of key antiviral mechanisms across species. This conservation is also crucial for interpreting preclinical xenotransplantation trials conducted in non‐human primates.

Analysis of gene expression in peripheral PBMCs from patients infected with adenovirus type 55 revealed that the expression levels of several interleukins—including IL‐18, IL‐36β, IL‐17 receptor, TNFSF10/11/14/15, and IL‐1α—correlated with disease severity. These findings provide valuable insights into the regulation of inflammatory genes during the progression of adenovirus infection [20].

These are just a few examples, but they illustrate both the power of OMICS analyses and the profound impact viral infections have on gene expression and metabolism. Such extensive changes can significantly interfere with the interpretation of OMICS data in the context of rejection in xenotransplantation. The situation is even more complicated if the animal to be tested and the control animal are infected with different viruses.

## 4 Conclusion

When extensive and sensitive screening for viral infections is conducted in parallel, OMICS analyses have the potential to be true game changers in xenotransplantation. It is crucial that viral infections of the donor and the recipient are carefully considered when applying OMICS approaches in this field. Moreover, as such methods also gain wider use in allotransplantation, the same level of caution is required to ensure reliable and accurate interpretation.

## Funding

The author has nothing to report.

## Conflicts of Interest

The author has no conflict of interest.
